# Patients with type 2 diabetes exhibit cognitive impairment with changes of metabolite concentration in the left hippocampus

**DOI:** 10.1007/s11011-015-9670-4

**Published:** 2015-04-16

**Authors:** Yue Wang, Xiao-yun Xu, Chun-hua Feng, Yuan-ling Li, Xia Ge, Gen-lin Zong, Yi-bin Wang, Bo Feng, Peng Zhang

**Affiliations:** Department of Neurology, East Hospital, Tongji University, Shanghai, China; Department of Neurology, Pudong New Area Zhoupu Hospital, Shanghai, China; Department of Radiology, East Hospital, Tongji University, Shanghai, China; Department of Endocrinology, East Hospital, Tongji University, Shanghai, China; Shanghai Pudong New Area Center for Disease Control & Prevention, Shanghai, China

**Keywords:** Type 2 diabetes mellitus, Cognitive function, Metabolite, Proton magnetic resonance spectroscopy

## Abstract

Type 2 diabetes mellitus (T2DM) is associated with cognitive dysfunction. Previous studies have reported the relationship between cerebral metabolite changes and glucose levels. However, the specific aspects of cognition that are affected by metabolic changes in T2DM- related cognitive impairment remain undetermined. In this study, 188 T2DM patients and 266 controls were recruited. Proton magnetic resonance spectra with a single voxel stimulated echo acquisition mode (STEAM) were acquired from the left hippocampus and the frontal lobe. Presence of T2DM negatively affected the scores of Mini-Mental State Examination (MMSE), sub-tests (i.e., attention and language) of MMSE, Montreal Cognitive Assessment (MoCA) according to the Beijing version, and sub-tests (i.e., visuospatial/executive reasoning, attention, and language) of MoCA, rather than the Wechsler Memory Scale – Revised in China (WMS-RC), and all memory sub-tests contained with the MMSE and MoCA frameworks. T2DM positively affected creatine and myoinositol peak areas from the left hippocampus, rather than metabolites in the left frontal lobe. Negative correlations were shown between the left hippocampal myoinositol levels and language scores, and between the left hippocampal creatine levels and visuospatial/executive scores in T2DM. These findings suggest that T2DM may be an independent risk factor for cognitive impairment. Further, the cognitive domains of visuospatial /executive reasoning, attention and language may be predominantly impaired in the early phases of T2DM-related cognitive impairment. In addition, left hippocampal myoinositol and creatine concentrations were associated with cognitive impairment in patients with T2DM.

## Introduction

Type 2 diabetes mellitus (T2DM) is associated with cognitive dysfunction and an increased risk of dementia. There is still uncertainty with regard the etiology, but vascular disease is likely to play a role (Biessels et al. 2008). There is growing interest in potential correlations between cognitive function and changes in cerebral metabolites in T2DM, since this disease presentation is a metabolic disorder.

At present, magnetic resonance spectroscopy (MRS) is the only available non-invasive method for detecting biochemical molecules within living organs (Vrenken et al. 2005). Recent studies have used MRS to reveal the relationship between changes in brain neurochemistry and glucose levels, such as decreased N-acetyl-aspartate (NAA)/creatine (Cr) ratios in the pons, and the left posterior parietal white matter of poorly controlled type 1 diabetes mellitus in children (Sarac et al. 2005) and in the frontal cortex of patients that presented with T2DM with higher glycosylated hemoglobin A1c (HbA1c) levels (>10 %) (Sahin et al. 2008). MRS has also been used to show increased myoinositol (mI) levels in the occipitoparietal grey matter of patients that presented with metabolic syndrome and normal cognition (Haley et al. 2010), and in cases of increased Cr in the thalamus of participants at risk of developing metabolic syndrome (Heikkilä et al. 2008), and in cases of increased choline-containing compounds (Cho)/Cr ratios in the left occipital lobe of T2DM patients (Modi et al. 2008). Other studies have used MRS to reveal the relationship between changes in neurochemistry and cognitive impairment. It was revealed that mI was increased and NAA was decreased in the occipital, parietal (Huang et al. 2001), and hippocampus (Watanabe et al. 2012) of Alzheimer’s disease patients. However, these studies did not investigate the specific aspects of cognition that reflected these metabolic changes in T2DM-related cognitive impairment.

In this study, we used neuropsychological scales and MRS in patients with T2DM to investigate the cognitive changes and neurochemical abnormalities in the left hippocampus and the left frontal lobe, which are both closely related to cognition in right-handed people, especially in the context of language message retention and memory, and executive reasoning processes (Smith and Jonides 1999; Schaefer et al. 2006; Watanabe et al. 2012; Jefferies 2013) that conform to neuropsychological scales. We evaluated the correlations between neuropsychological scales and levels of specific metabolites.

## Methods

### Participants

All participants were recruited consecutively at the out-patient department of endocrinology and neurology of Shanghai East Hospital affiliated to Tongji University between September 2009 and February 2014. Recruited patients underwent comprehensive diagnostic evaluation, including demographic parameters such as age, gender, educational status, body mass index (BMI), personal histories of hypertension, presence of T2DM, cardiovascular disease, presence of hyperlipidemia, and lifestyle risk factors including smoking habits, neurological and psychiatric examinations, neuropsychological testing, basic laboratory tests, and clinical laboratory findings such as fasting blood glucose levels, glycosylated hemoglobin A1c (HbA1c) and brain magnetic resonance imaging (MRI).

Inclusion criteria of the T2DM group included: (1) T2DM that was diagnosed in accordance with the diagnostic criteria of the American Diabetes Association, 1997, (2) timespan of T2DM of 3–10 years, (3) right handedness, (4) urine ketone negative, and (5) an Activity of Daily Living scale (ADL) score ≤ 16, a Global Deterioration Scale (GDS) score ≤3, and a Hamilton Depression scale (HAMD) score ≤7. Inclusion criteria of the control group included: (1) non-T2DM, (2) right handedness, and (3) an ADL score ≤ 16, a GDS score ≤3, and an HAMD score ≤7. The exclusion criteria were medical histories of stroke or other major neurological disease or condition, evidence of psychiatric disorders (e.g., schizophrenia, and bipolar disorder, etc.), substance abuse (e.g., alcohol, drugs, or other), and metabolic encephalopathy (e.g., hepatic encephalopathy, and pulmonary encephalopathy).

The protocol was approved by the local ethics committees of the Shanghai East Hospital Affiliated to Tongji University and the study was operated in accordance with the World Medical Association’s Declaration of Helsinki. All of the participants provided written and informed consent before enrollment. In total, 188 patients that had presented with T2DM and 266 participants with non-T2DM were recruited to this study. All diagnosed medical conditions had been treated in these participants.

### Assessments

#### Blood biochemistry

Fasting blood concentrations of glucose, HbA1c, routine urinalysis, total triglycerides, total cholesterol, high density lipoprotein (HDL)-cholesterol levels, and low density lipoprotein (LDL)-cholesterol levels were detected by standard enzymatic assays. Radial artery blood pressure was measured by a standard blood pressure monitor (VP-2000, Colin Medical Technology Corporation, Japan) after at least 10 min of whole body rest.

#### Neuropsychological evaluation

Cognitive assessments included the Mini-mental State Examination (MMSE), Montreal Cognitive Assessment (MoCA) – Beijing version, and Wechsler Memory Scale - Revised in China (WMS-RC) (Gong. 1989). Cognitive evaluation and blood biochemical measurements were also performed on the same day. The participants were not fasting during the cognitive evaluation.

The MMSE consisted of orientation, immediate memory, attention, delayed memory, language (i.e., naming, repeating, auditory comprehension, reading, and writing skills), and visual-spatial ability, for a maximum total score of 30. The MoCA tests included analysis of visuospatial/executive reasoning, naming, attention, memory, language, abstraction, and orientation skills, which were aggregated for a maximum score of 30. WMS-RC tests included long-term memory (i.e., personal experience, orientation, and digit order), short- term memory (i.e., visual recognition, visual reproduction, picture recollection, associative learning skills, touch test, and comprehension memory skill), and immediate-term memory (i.e., digit span). A normal range for the memory quotient (MQ) is 90–109.

All 454 participants were assessed by MMSE and MoCA, and among them 98 participants were assessed by WMS-RC. All tests were administered and scored by a professionally trained operator in neuropsychometric testing.

#### MRI and ^1^H-MRS

Both MRI and proton magnetic resonance spectroscopy (^1^H-MRS) were performed on a 3.0 Tesla apparatus (Philips Intera Achieva, Netherlands) using a standard head coil suited for MRI and MRS. The protocol for structural MRI included axial T2 weight imaging (T2WI), sagittal T2WI, and hippocampal oblique coronal T2WI with repetition time (TR)/echo time (TE) = 5500/130 with a slice thickness of 6 mm and a gap of 1 mm.

**Fig. 1:**
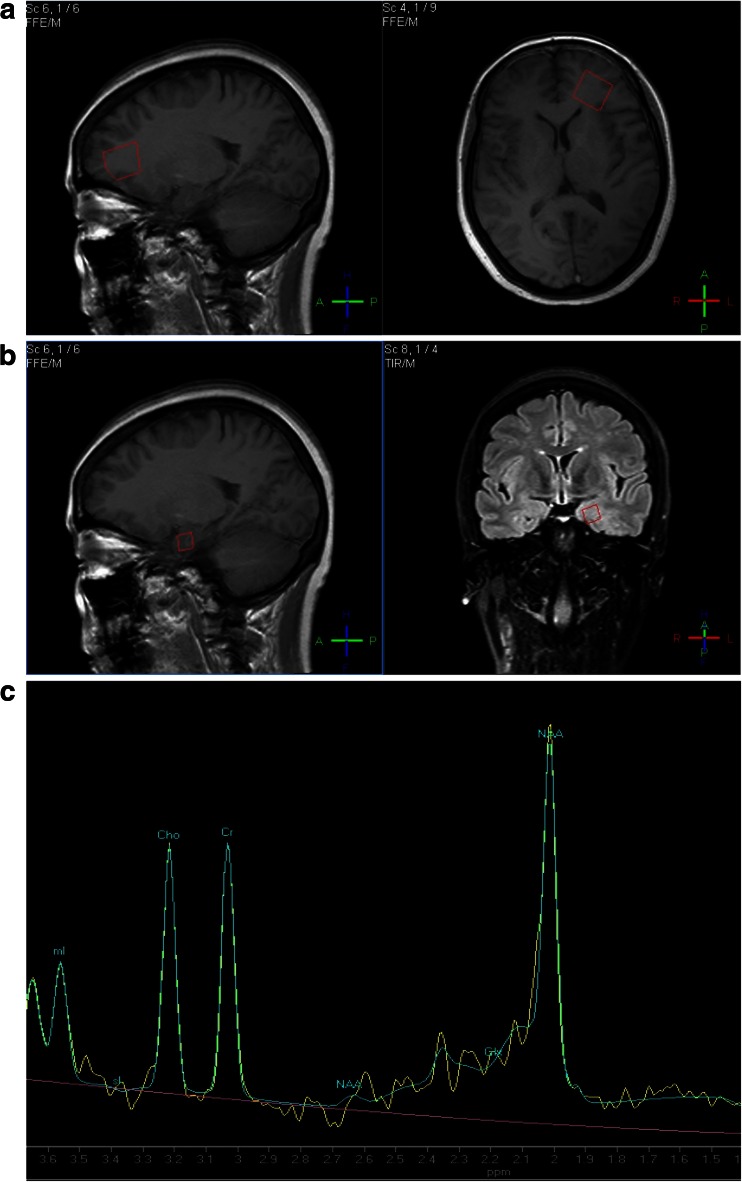
The region of interest: volume 20 mm^3^ from the left frontal lobe (**a**) and volume 10 mm^3^ from the left hippocampus (**b**), and a left hippocampal spectrum of a participant with type 2 diabetes mellitus (**c**)

In order to measure the mI concentrations as a measure of the metabolism of short T2, and to improve the sensitivity and resolution, we chose stimulated echo acquisition mode (STEAM) analysis on a 3.0 Tesla apparatus. ^1^H-MRS parameters were as follows: TE = 9.4 ms, TR = 2000 ms, 128 excitations, acquire time (TA) = 4 min 48 s, a volume of 10 mm^3^ from the left hippocampus and 20 mm^3^ from the left frontal lobe (Fig. 1). The chosen volumes covered the region of interest (ROI) as completely as possible without including the cerebral spinal fluid (CSF) or other regions. Smaller regions than those shown above, would have caused deviations due to the slight movement that occurs with breathing and regular heartbeats.

Cerebral structural changes such as lacunar cerebral infarction, leukoaraiosis, and encephalatrophy were observed visually and classified as yes or no. Spectral peak areas corresponding to the metabolites NAA (2.0 ppm), Cho (3.2 ppm), Cr (3.02 ppm), and mI (3.56 ppm) were quantified in the frequency domain, fitting for frequencies, and areas and line widths with a Gaussian line shape using in-house software. All images were acquired by a professional radiology technician, which was conducted in a double-blind data acquisition method. The participants were not fasting during the ^1^H-MRS procedure. Further, the interval between cognitive evaluation and imaging was less than three days. The tests performed were conducted in the same sequence for all participants.

### Statistical analyses

Data were analyzed using the Statistical Package for Social Sciences (SPSS version 17.0; SPSS, Chicago, IL, USA). All comparisons of the demographic data were performed by *t* tests and χ2 tests. Multiple liner regression analyses calculated the associations of the neuropsychological scales and the variables of metabolite concentrations found in patients presenting with T2DM. Partial correlation coefficients were calculated to reveal associations between the variables of metabolite concentrations and neuropsychological scales. All values described in the text and tables were mean and standard deviation unless stated otherwise. A two-tailed alpha level of *P* < 0.05 was the criterion for statistical significance.

## Results

### Demographics

A total of 454 participants were in the final sample analysis, of which 188 patients were in the T2DM patient group and 266 were in the control group. The duration of diabetes was calculated as 76.16 ± 37.98 months in the T2DM group. The demographic details and clinical characteristics of the subjects are summarized in Table 1.

**Table 1:** Demographics and clinical characteristics in T2DM group and control group

		T2DM group	Control group	*p*
Gender	Men (n)	95	128	0.64
	Women (n)	93	138	
Age (year)		60.97 ± 9.05	60.50 ± 10.85	0.59
Educational status (year)		9.32 ± 3.80	10.18 ± 3.95	0.02
Fasting blood glucose(mmol/L)		8.65 ± 4.01	4.99 ± 0.65	<0.001
HbA1c (%)		8.45 ± 2.19	5.70 ± 0.29	<0.001
Hypertension (n)		134(71.28 %)	124(46.62 %)	<0.001
Systolic pressure(mmHg)		136.76 ± 11.15	130.17 ± 10.58	<0.001
Diastolic pressure(mmHg)		87.02 ± 6.04	84.13 ± 4.90	<0.001
Hyperlipidemia (n)		53(28.19 %)	52(19.55 %)	0.04
Cholesterol(mmol/L)		4.76 ± 1.13	4.82 ± 1.07	0.66
Triglycerides(mmol/L)		2.05 ± 1.74	1.61 ± 1.10	0.02
HDL(mmol/L)		1.18 ± 0.35	1.35 ± 0.49	0.001
LDL(mmol/L)		3.00 ± 0.88	3.07 ± 0.98	0.52
Cardiovascular disease (n)		60(31.91 %)	56(21.05 %)	0.01
Smoking habits (n)		72(38.30 %)	72(27.07 %)	0.01
BMI		24.40 ± 2.74	23.19 ± 3.01	<0.001
Cerebral structural changes (n)		156(82.98 %)	209(78.57 %)	0.28
MMSE		25.09 ± 4.29	26.06 ± 3.81	0.01
MoCA		18.81 ± 5.63	20.76 ± 5.02	<0.001
Memory quotient		78.24 ± 21.92	88.50 ± 18.76	0.08
Left hippocampus	NAA	0.072 ± 0.088	0.064 ± 0.079	0.15
	Cr	0.066 ± 0.076	0.049 ± 0.053	0.01
	Cho	0.055 ± 0.091	0.053 ± 0.055	0.18
	mI	0.064 ± 0.071	0.044 ± 0.058	0.02
Left frontal lobe	NAA	0.348 ± 0.350	0.353 ± 0.365	0.51
	Cr	0.234 ± 0.239	0.237 ± 0.265	0.41
	Cho	0.206 ± 0.201	0.199 ± 0.268	0.41
	mI	0.136 ± 0.167	0.127 ± 0.266	0.33

### Cognitive function and metabolite concentrations

The *t*-tests showed that the T2DM group had significantly lower scores for MMSE and MoCA than did the control group (all *p* < 0.05). However, there were no significant differences in MQ scores between groups (Table 1). Multiple liner regression analyses, using the scores of MMSE, sub-tests of MMSE, MoCA, and sub-tests of MoCA as dependent variables and age, gender, educational status, smoking habits, BMI, presence of T2DM, hypertension, cardiovascular disease, hyperlipidemia, and cerebral structural changes as independent variables in all participants, evaluated the associations. Tables 2–3 showed that T2DM negatively affected the scores of MMSE, the sub-tests (i.e., attention and language) of MMSE, MoCA, and sub-tests (i.e., visuospatial/executive reasoning, attention, and language skills) of MoCA (all *p* < 0.05). T2DM did not significantly affect all memory subtests of MMSE and MoCA.

**Table 2:** Multiple linear regression analyses of MMSE, subtests of MMSE, and interference factors for cognitive changes in all participants

	MMSE	Subtests of MMSE					
		Orientation	Immediate memory	Attention	Delayed memory	Language	Visual-spatial ability
Constant	24.567	9.176	2.780	4.944	2.337	4.796	0.564
Gender	0.524	0.071	0.044	−0.102	0.095	0.334^a^	0.063
Age	−0.093^b^	−0.023^b^	−0.006^a^	−0.020^b^	−0.026^b^	−0.011	−0.006^a^
Educational status	0.426^b^	0.071^b^	0.028^b^	0.070^b^	0.051^b^	0.168^b^	0.040^b^
Hypertension	0.738^a^	0.162	0.066	0.178	0.143	0.230^a^	0.031
Hyperlipidemia	−0.220	0.208	0.025	−0.237	−0.107	−0.121	0.027
Cardiovascular disease	0.271	−0.009	−0.045	0.189	0.062	0.182	−0.041
Smoking habits	0.497	0.066	−0.011	0.105	−0.031	0.298^a^	0.101
Cerebral structural changes	−0.700	−0.113	−0.003	−0.377^a^	−0.028	−0.271	−0.007
BMI	0.089	0.026	0.003	0.001	0.021	0.030	0.003
T2DM	−0.784^a^	−0.098	−0.009	−0.257^a^	−0.007	−0.328^b^	−0.071

a *p* < 0.05

b *p* < 0.01

**Table 3:** Multiple linear regression analyses of MoCA, subtests of MoCA, and interference factors for cognitive changes in all participants

	MoCA	Subtests of MoCA						
		Visuospatial/executive	Naming	Attention	Language	Abstraction	Memory	Orientation
Constant	21.612	2.879	1.284	4.918	0.727	0.352	2.958	6.478
Gender	0.195	−0.027	−0.241^a^	0.027	0.179	−0.079	0.405^a^	0.042
Age	−0.165^b^	−0.023^b^	−0.006	−0.025^b^	−0.016^b^	−0.010^a^	−0.056^b^	−0.017^b^
Educational status	0.600^b^	0.145^b^	0.087^b^	0.129^b^	0.073^b^	0.072^b^	0.109^b^	0.036^b^
Hypertension	0.536	0.075	−0.063	0.145	0.063	−0.008	0.266	0.098
Hyperlipidemia	−0.114	−0.049	0.071	−0.097	−0.023	−0.007	−0.213	0.154
Cardiovascular disease	0.636	0.242	0.143	0.219	0.064	0.013	−0.058	0.001
Smoking habits	0.374	0.184	0.167	0.039	0.087	−0.022	−0.111	0.055
Cerebral structural changes	−1.250^a^	−0.374^a^	−0.089	−0.407^b^	−0.132	−0.015	−0.349	−0.126
BMI	0.132	0.017	0.027	0.015	0.036^b^	0.019	0.038	−0.014
T2DM	−1.629^b^	−0.397^b^	−0.005	−0.335^b^	−0.302^b^	−0.146	−0.235	−0.108

a *p* < 0.05

b *p* < 0.01

Using ^1^H-MRS, the absolute concentrations of NAA, Cho, Cr, and mI in the left hippocampus and left frontal lobe were obtained. Multiple linear regression analyses that used the absolute concentrations of NAA, Cho, Cr, and mI in the left hippocampus and left frontal lobe as dependent variables, and age, gender, smoking habits, BMI, T2DM, hypertension, cardiovascular disease, hyperlipidemia, and cerebral structural changes as independent variables in all participants, were employed to evaluate the putative associations. Table 4 showed that T2DM positively affected the concentrations of Cr and mI in the left hippocampus (all *p* < 0.05). Further, T2DM did not significantly affect the left frontal lobe NAA, Cho, Cr, and mI levels and the left hippocampal NAA and Cho levels.

**Table 4:** Multiple linear regression analyses of brain metabolites in the left hippocampus and left frontal lobe, and interference factors for metabolite changes in all participants

	Left hippocampus				Left frontal lobe			
	NAA	Cr	mI	Cho	NAA	Cr	mI	Cho
Constant	0.182	0.046	0.154	0.170	0.276	−0.074	−0.594	4.801
Gender	0.027	0.077	0.034	0.199	−0.022	0.025	0.026	0.422
Age	−0.002	−0.001	−0.002	−0.002	0.005	0.004	0.011	−0.049
Hypertension	−0.022	−0.085	−0.062^b^	−0.188	0.009	0.057	0.170	0.946
Hyperlipidemia	0.020	0.059	0.011	0.232^a^	−0.060	−0.057	−0.184	−0.495
Cardiovascular disease	0.023	0.120^b^	0.080^b^	0.246^a^	−0.110	−0.098	−0.286	−0.303
Smoking habits	0.011	0.081	0.029	0.045	−0.007	0.002	0.282	−0.159
Cerebral structural changes	−0.063^a^	−0.007	0.027	−0.046	−0.061	0.035	0.017	0.865
BMI	−0.004	−0.002	−0.003	−0.010	0.000	0.004	0.010	−0.111
T2DM	0.042	0.153^b^	0.057^b^	0.166	−0.031	−0.039	−0.206	−0.436

a *p* < 0.05

b *p* < 0.01

### Correlation of the left hippocampal Cr and mI concentrations with neuropsychological scales

Partial correlation coefficients were calculated to reveal the correlations of the left hippocampal Cr and mI concentrations with the following: the psychological scales, adjusted age, gender, educational status, smoking habits, BMI, HbA1c, hypertension, cardiovascular disease, hyperlipidemia, changes in cerebral structure, and other metabolic concentrations in the left frontal lobe and left hippocampus. Table 5 showed that there was a significant negative correlation between the levels of left hippocampal mI and the language scores of MoCA in patients that presented with T2DM. There was also a significant negative correlation between the left hippocampal Cr concentration and the visuospatial/executive scores of MoCA in patients that had presented with T2DM. Table 5 also showed that there were no significant correlations between the left hippocampal Cr and the mI concentrations and neuropsychological scales in the control group.

**Table 5:** Correlation of the left hippocampal Cr and mI concentrations with neuropsychological scales

Neuropsychological scale	Subtests of scale	T2DM group		Control group	
		mI	Cr	mI	Cr
MoCA		0.058	−0.118	0.089	0.070
	Visuospatial/executive	0.184	−0.233^a^	0.036	0.066
	Attention	−0.008	0.134	0.090	−0.079
	Language	−0.233^a^	0.152	0.091	0.008
MMSE		0.040	0.074	0.078	−0.003
	Attention	0.011	0.227	0.028	0.094
	Language	0.037	−0.059	0.090	−0.131

a *p* < 0.05

## Discussion

Clinically, individuals that present with T2DM commonly have a cluster of accompanying interrelated metabolic diseases including hypertension, cardiovascular disease, hyperlipidemia, and cerebral structural changes like leukoaraiosis (Bastos-Leite et al. 2008; van Harten et al. 2006), cortical and subcortical atrophy (Musen et al. 2006; Wessels et al. 2006), which have all been linked to cognitive decline (Gatto et al. 2008; Yaffe et al. 2009). In this study, we adjusted the multiple interfering factors of cognitive changes in an attempt to observe T2DM-related cognitive impairment in the clinical setting.

We found that T2DM negatively affected the scores of MMSE, MoCA, sub-tests (attention and language skills) of MMSE, and sub-tests (i.e., visuospatial/executive, attention, and language skills) of MoCA, rather than the scores of MQ and all memory sub-tests of MMSE and MoCA. These results suggested that T2DM-related cognitive impairment occurred in our study, and were early cognitive impairments, in part because one of the inclusion criteria was an ADL score ≤ 16, an HAMD score ≤7, and a GDS score ≤3. Thus, T2DM might be an independent risk factor for cognitive impairment, and the cognitive domains of visuospatial/ executive reasoning, and attention and language skills might be predominantly impaired in the early phases of T2DM-related cognitive impairment, rather than the cognitive function of memory.

NAA has been proposed as a marker of neuronal density and viability (Moffett et al. 2007), Cho is involved in membrane breakdown (Gujar et al. 2005), Cr is involved in energy metabolism, and increased Cr has previously been shown to be related to increased oxidative metabolism in both neurons and glial cells (Ross and Bluml 2001), and mI has been interpreted as a marker of gliosis, an organic osmolyte and a precursor in the synthesis of the second messenger inositol tri-phosphate (Fisher et al. 2002).

T2DM positively affected the levels of Cr and mI in the left hippocampus, and there was no significant relationship between T2DM and the left hippocampal NAA and Cho in this study, which suggested that neuronal density was unchanged, glial cell membrane turnover was intact and the left hippocampal Cr and mI levels were increased in T2DM. An animal study showed that the concentrations of mI and Cr in the hippocampus of Zucker Diabetic Fatty rats were increased (van der Graaf et al. 2004). In addition, insulin receptors in the hippocampal neurons are highly concentrated (Park 2001), suggesting that the synthesis of energy in the hippocampus may be abnormal, resulting from insulin resistance due to the glucose metabolism disorder that characterizes T2DM. The increased Cr may therefore indicate an increased need for an energy buffering capacity in the left hippocampus in the setting of T2DM. In addition, elevated left hippocampal mI in T2DM may be interpreted as a sign of gliosis, which we have previously reported (Li et al. 2011), and disturbance in intracellular second messenger transmission, caused by insulin resistance in T2DM.

Our study also showed that left hippocampal mI concentrations were negatively correlated with language scores and the left hippocampal Cr concentrations were negatively correlated with visuospatial/executive reasoning scores in T2DM. There were strong correlations between the mI concentrations and the number of neuritic plaques and neurofibrillary tangles in patients with Alzheimer’s disease (Ross and Sachdev. 2004). In addition, elevated concentrations of mI have been reported in the bilateral hippocampi of Alzheimer’s disease patients. Furthermore, left hippocampal mI concentrations were associated with cognitive dysfunction commonly observed in patients with Alzheimer’s disease (Watanabe et al. 2012). Our results suggested that left hippocampal myoinositol and creatine levels were associated with cognitive impairment in T2DM patients.

In the present study, no significant relationships were found between any metabolite level in the left frontal lobe and T2DM. A similar result was found by Modi et al. (2008) who reported that Cho/Cr and NAA/Cr ratios showed no differences in the left frontal white matter when comparing T2DM patients and normal controls. In this study, T2DM affected changes in metabolites in the left hippocampus rather than the left frontal lobe, which might suggest that metabolite changes were not the same in different cerebral regions of subjects that presented with T2DM with a duration that ranged from 3 to 10 years. The key reason for this is that the subjects that were studied in this article had undergone glycemic control.

Though the present study is limited by multiple interference factors of changes in neuro- cognitive function, the results clearly gave insights into the metabolite changes that were associated with T2DM-related cognitive impairment. Further studies are needed to demonstrate whether or not differential medical therapies affect the metabolic changes that are associated with T2DM-related cognitive impairment.

In conclusion,T2DM might be an independent risk factor for cognitive impairment, and might lead to changes in left hippocampal metabolism. Cognitive domains of visuospatial /executive, and attention and language skills might be predominantly impaired in early T2DM-related cognitive impairment. Increased left hippocampal myoinositol and creatine levels that were measured by ^1^H-MRS might reflect cognitive impairment in T2DM patients.
